# Substitution of fluorine in M[C_6_F_5_BF_3_] with organolithium compounds: distinctions between O- and N-nucleophiles

**DOI:** 10.3762/bjoc.13.69

**Published:** 2017-04-12

**Authors:** Anton Yu Shabalin, Nicolay Yu Adonin, Vadim V Bardin

**Affiliations:** 1 G.K. Boreskov Institute of Catalysis, SB RAS, Acad. Lavrentjev Ave. 5, Novosibirsk, 630090, Russian Federation; 2 Novosibirsk State University, Pirogova str. 2, Novosibirsk, 630090, Russian Federation; 3 N.N. Vorozhtsov Novosibirsk Institute of Organic Chemistry, SB RAS, Acad. Lavrentjev Ave. 9, Novosibirsk, 630090, Russian Federation

**Keywords:** C-nucleophile, NMR spectroscopy, nucleophilic substitution, pentafluorophenyltrifluoroborate

## Abstract

Borates M[C_6_F_5_BF_3_] (M = K, Li, Bu_4_N) react with organolithium compounds, RLi (R = Me, Bu, Ph), in 1,2-dimethoxyethane or diglyme to give M[4-RC_6_F_4_BF_3_] and M[2-RC_6_F_4_BF_3_]. When R is Me or Bu, the nucleophilic substitution of the fluorine atom at the *para* position to boron is the predominant route. When R = Ph, the ratio M[4-RC_6_F_4_BF_3_]/M[2-RC_6_F_4_BF_3_] is ca. 1:1. Substitution of the fluorine atom at the *ortho* position to boron is solely caused by the coordination of RLi via the lithium atom with the fluorine atoms of the BF_3_ group. This differs from the previously reported substitution in K[C_6_F_5_BF_3_] by O- and N-nucleophiles that did not produce K[2-NuC_6_F_4_BF_3_].

## Introduction

**Scheme 1:**
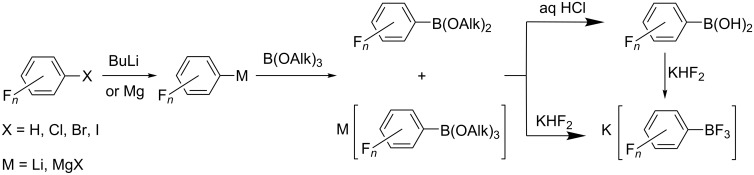
Preparation of polyfluoroorganotrifluoroborates.

Organoborates M[RBX_3_] (X = OAlk, F) are widely used in various fields of chemistry [1–12]. Their polyfluorinated analogues M[R_F_BX_3_] have been used as starting reagents in the synthesis of compounds of hypervalent bromine [13], iodine [14–16] and xenon [17–21]. Over the last 15 years, we reported the successful application of polyfluorinated organoborates K[RC_6_F_4_BF_3_], K[C_6_F_5_B(OMe)_3_] and K[CF_2_=CFBF_3_] as boron-containing reagents in the Pd-catalyzed cross-coupling reactions with C-electrophiles [22–27]. Nowadays a common approach to these compounds is based on the transformation of polyfluoroarenes under the action of appropriate reagents into the corresponding organometallic derivatives followed by treating them with suitable boron-containing electrophiles (Scheme 1) [28–29]. In order to further expand this powerful tool for the introduction of polyfluorinated building blocks into organic molecules, the synthesis of a series of polyfluoroaryltrifluoroborates with substituents different from fluorine atoms is desirable.

However, practical application of this route requires the corresponding starting substances Ar_F_X, which in many cases are expensive. An alternative approach is based on modification of easily available potassium pentafluorophenyltrifluoroborate (**1-K**) and we carried out systematic research in this field. Thus, K[C_6_F_5_BF_3_] was converted into K[2,3,4,5-C_6_HF_4_BF_3_] using NiCl_2_·6H_2_O and Zn in the presence of bpy in aprotic polar solvents (DMF, DMA or NMP) [30]. At present the main direction is the study of the substitution of aromatically bonded fluorine atoms in K[C_6_F_5_BF_3_] with nucleophiles of different nature. The salts K[4-ROC_6_F_4_BF_3_] (R = Me, Et, Pr, iPr, Bu, *t*-Bu, PhCH_2_, CH_2_=CHCH_2_, Ph) were prepared by alkoxydefluorination of K[C_6_F_5_BF_3_] with the corresponding O-nucleophiles RONa or ROK [31]. The nucleophilic substitution of a fluorine atom in K[C_6_F_5_BF_3_] with sodium (potassium) azolides in polar aprotic solvent (DMF, DMSO) at 100–130 °C resulted in K[4-R_2_NC_6_F_4_BF_3_] (R_2_N = pyrrolyl, pyrazolyl, imidazolyl, indolyl, and benzimidazolyl) with 74–93% isolated yield. In contrast, sodium morpholinide and sodium diethylamide did not react with K[C_6_F_5_BF_3_] under the same conditions. The attempted preparation of K[4-R_2_NC_6_F_4_BF_3_] (R_2_N = morpholinyl, Et_2_N) using an excess of dialkylamine as well as morpholine and K_2_CO_3_ leads to C_6_F_5_H and dialkylaminotetrafluorobenzene [32]. Additional experiments on the competitive nucleophilic substitution of 2,3,4,5,6-pentafluorobiphenyl (model substrate) with sodium indolide and sodium morpholinide (DMF, 130 °C, 4 h) showed the kinetic reason of this phenomenon: the first nucleophile reacts with the substrate much faster than the second one. In the case of K[C_6_F_5_BF_3_] this leads to the formation of C_6_F_5_H (byproduct) rather than the formation of K[4-R_2_NC_6_F_4_BF_3_] due very slow aminodefluorination with NaNR_2_ [32].

Being interested in a wide series of polyfluoroaryltrifluoroborates, we investigated possible reaction routes from M[C_6_F_5_BF_3_] (M = K, Li and Bu_4_N) to alkyl-, alkynyl- and aryltetrafluorophenyltrifluoroborates using the nucleophilic substitution with some organolithium compounds. The obtained results were compared with previously reported data [31–32].

## Results

### Reactions with MeLi

**Scheme 2:**
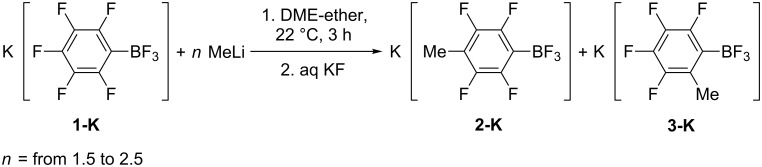
Interaction of K[C_6_F_5_BF_3_] (**1-K**) with methyllithium (byproducts of hydrodeboration are not depicted).

An addition of MeLi (1.5 equiv) in ether to a solution of K[C_6_F_5_BF_3_] (**1-K**) in DME causes precipitation of a white solid. Stirring of the suspension at 22 °C for 3 h with subsequent treatment with aqueous KF gave potassium 4-methyltetrafluorophenyltrifluoroborate, K[4-MeC_6_F_4_BF_3_] (**2-K**) and potassium 2-methyltetrafluorophenyltrifluoroborate, K[2-MeC_6_F_4_BF_3_] (**3-K**) (1:0.13) besides unreacted **1-K** (total conversion 51%) (Table 1, entry 1). A prolongation of the reaction time up to 6 h has no effect on composition of products (Table 1, entry 2). In the presence of a large excess of the nucleophile (2.5 equiv of MeLi) conversion of **1-K** increases up to 85% (Table 1, entry 3) and 100% (3.6 equiv of MeLi) (Table 1, entry 4). When the reaction was performed at 43–47 °C for 3 h, the conversion of **1-K** was 83%, but the yield of borate **2-K** was lower because of side reactions (mainly, hydrodeboration) (Table 1, entry 5). The reflux of **1-K** with 2.0 equiv of MeLi in DME–ether for 5 h gave **2-K** and **3-K** besides a small quantity of **1-K** (Table 1, entry 6) (Scheme 2).

**Table 1:** Reaction of K[C_6_F_5_BF_3_] (**1-K**) with methyllithium (22 °C, 3 h).

entry	**1-K**, mg (mmol)	DME, mL	MeLi, mL (mmol)	conversion of **1-K**, %^a^	selectivity, %^a^	

					**2-K**	**3-K**

1	97 (0.35)	3	1.5 (0.54)	51	83	11
2	115 (0.41)^b^	3	1.7 (0.61)	59	79	8
3	108 (0.39)	4	2.7 (0.97)	85	73	9
4	276 (1.0)	3	10 (3.6)	100	55	5
5	113 (0.41)^c^	3	2.7 (0.97)	83	15	—
6	170 (0.62)^d^	2	2 (1.28)	92	39	4

^a^from ^19^F NMR data; ^b^duration 6 h; ^c^at 43–47 °C. Reaction mixture contained C_6_F_5_H (0.03 mmol, selectivity 9%) and 2,3,5,6-tetrafluorotoluene (**4**) (0.05 mmol, selectivity 15%) and minor unknown components; ^d^reaction mixture was refluxed for 5 h (53–55 °C, bath); the filtrate contained unknown minor products besides the borates **1-K**, **2-K**, and **3-K**.

The use of 3.9 equiv of the nucleophile and reflux of the suspension for 1 h led to the total consumption of **1-K** but the desired aryltrifluoroborates were not obtained. Instead, a mixture of many unknown products forms in which a small amount 2,3,5,6-tetrafluorotoluene (**4**) was identified. Treatment of these products with aqueous KF increased the content of **4** and led to the appearance of C_6_F_5_H and 2,3,4,5-tetrafluorotoluene (**5**) (^19^F NMR), which may be attributed to hydrodeboration of unrecognized arylboron compounds.

### Reactions with BuLi

**Scheme 3:**
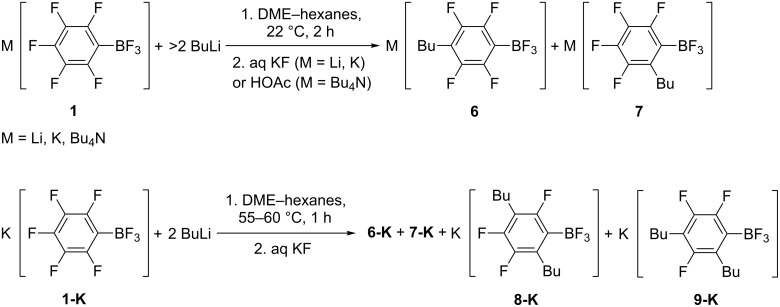
Interaction of M[C_6_F_5_BF_3_] (**1-M**) with butyllithium (byproducts of hydrodeboration are not depicted).

In general, reactions of **1-K** with BuLi proceed as reactions with MeLi although the precipitation was not observed. The reaction of BuLi (2 equiv) with **1-K** in DME–hexanes at 22 °C for 2 h and the subsequent treatment of the reaction mixture with aqueous KF gave potassium 4-butyltetrafluorophenyltrifluoroborate (**6-K**) and potassium 2-butyltetrafluorophenyltrifluoroborate (**7-K**) (molar ratio 1:0.18) (Table 2, entry 1). In the presence of a larger excess of BuLi the quantity of **7-K** reduced to 1:0.10, presumably because of further substitution (Table 2, entry 2). Heating the reaction mixture at 55–60 °C for 1 h leads to substitution of two fluorine atoms with the formation of potassium 2,5-dibutyltrifluorophenyltrifluoroborate (**8**) and potassium 2,4-dibutyltrifluorophenyltrifluoroborate (**9**) (minor) besides **6-K** and **7-K** (major) (Table 2, entry 3). Using Li[C_6_F_5_BF_3_] (**1-Li**) or [Bu_4_N][C_6_F_5_BF_3_] (**1-N**) gives the corresponding salts **6-Li**, **7-Li** and **6-N**, **7-N** (Table 2, entries 4–6) (Scheme 3). An analytically pure sample of **6-K** was isolated by crystallization of a mixture of **6-K** and **7-N** from MeCN.

**Table 2:** Reaction of M[C_6_F_5_BF_3_] (**1-M**) with butyllithium (22 °C, 2 h).

entry	M	**1-M**, mg (mmol)	DME, mL	BuLi, mL (mmol)	conversion of **1-M**, %^a^	selectivity, %^a^	

						**6-M**	**7-M**

1	K	94 (0.34)	4	0.3 (0.72)	100	65	12
2	K	279 (1.0)	10	1.2 (2.8)	100	65	7
3	K	162 (0.59)^b^	3	0.5 (1.2)	100	71	5
4	Li	418 (1.50)	6	1.3 (3.1)	97	68	10
5	Bu_4_N	200 (0.41)	3	0.4 (0.96)	56	52	35
6	Bu_4_N	429 (0.90)	6^c^	1.0 (2.5)	80	74	25

^a^from ^19^F NMR data; ^b^at 55–60 °C (bath) for 1 h; the reaction mixture contained K[2,5-Bu_2_C_6_F_3_BF_3_] (**8**) (0.02 mmol, selectivity 3%) and K[2,4-Bu_2_C_6_F_3_BF_3_] (**9**) (0.03 mmol, selectivity 5%); ^c^in diglyme.

### Reactions with PhLi

**Scheme 4:**
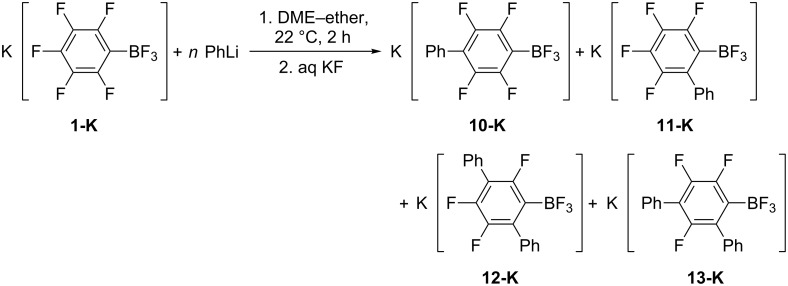
Interaction of K[C_6_F_5_BF_3_] (**1-K**) with phenyllithium (byproducts of hydrodeboration are not depicted).

The addition of PhLi in ether to a solution of **1-K** in DME leads to the formation of a precipitate similar to that in the reaction with MeLi. Contrary to the nucleophilic alkylation, the use of equimolar amounts of phenyllithium leads to complete consumption of **1-K** and the formation of potassium 4-phenyltetrafluorophenyltrifluoroborate (**10-K**), potassium 2-phenyltetrafluorophenyltrifluoroborate (**11-K**) and admixtures of potassium 2,5-diphenyltrifluorophenyltrifluoroborate (**12-K**) and potassium 2,4-diphenyltrifluorophenyltrifluoroborate (**13-K**) (Table 3, entry 1). The reaction of **1-K** with a subequimolar amount of phenyllithium (0.8 equiv) in DME–ether at 22 °C for 2 h gave a mixture of starting borate, and small amounts of **10-K** and **11-K** (Table 3, entry 2). Prolongation of the reaction time up to 6 h increases yields of **10-K** and **11-K** but **1-K** remains a predominant component (Table 3, entry 3). When **1-K** reacts with a three-fold excess of PhLi, the yields of monoarylated borates **10-K** and **11-K** become equal to that of diarylated borates **12-K** and **13-K** (Table 3, entry 4). In the presence of large excess of nucleophile borates **12-K** and **13-K** are the main products while compounds **10-K** and **11-K** were present in trace amounts (Table 3, entry 5, Scheme 4). Some unknown by-products were also formed.

**Table 3:** Reaction of K[C_6_F_5_BF_3_] (**1-K**) with phenyllithium (22 °C, 2 h).

entry	**1-K**, mg (mmol)	DME, mL	PhLi, mmol	conversion of **1-K**, %^a^	selectivity, %^a^			

					**10-K**	**11-K**	**12-K**	**13-K**

1	94 (0.34)	3	0.36	100	21	38	3	15
2	131 (0.48)	3	0.39	33	19	25		
3^b^	137 (0.50)	5	0.42	52	42	38		
4	124 (0.45)	5	1.40	100	27	20	4	40
5	95 (0.34)	3	2.10	100	6		6	53
6^c^	132 (0.48)	5	0.77	96	35	33		4

^a^from ^19^F NMR data; ^b^6 h; ^c^at 37–40 °C for 1 h; reaction mixture contained 2,3,5,6-tetrafluorobiphenyl (**14,** 0.01 mmol, selectivity 2%) and 2,3,4,5-tetrafluorobiphenyl (**15**, 0.02 mmol, selectivity 4%); borates **1**, **10**, **11** and **13** are lithium salts.

When **1-K** reacts with an excess of PhLi (1.6 equiv) at 37–40 °C for 1 h, the supernatant after treatment with aqueous KF contains **10-K** and **11-K** besides traces of **1-K** and **13-K** (Table 3, entry 6). Additionally, 2,3,5,6-tetrafluorobiphenyl (**14**) and 2,3,4,5-tetrafluorobiphenyl (**15**) were found.

### Reactions with PhC≡CLi

Attempts to involve **1-K** in the reaction with PhC≡CLi failed. Stirring the reagents in DME–ether solution at 22 °C for 17 h leads to recovery of borate **1-K**. The same result was obtained at 40 °C (2 h) and under reflux (58 °C, bath) for 5 h. It should be noted that in all cases **1-K** was recovered unchanged, e.g., no side reactions occurred.

In addition to identifying the reaction products by NMR spectroscopy, we confirmed their constitution by using the hydrodeboration reaction. This method consists in replacement of the BF_3_ group in polyfluoroaryltrifluoroborates by hydrogen in alcohol at elevated temperature and obtaining the corresponding polyfluoroarenes in high yields. The latter are more simple substances and available for analysis by NMR spectroscopy, GC–MS and HRMS methods [33].

**Scheme 5:**
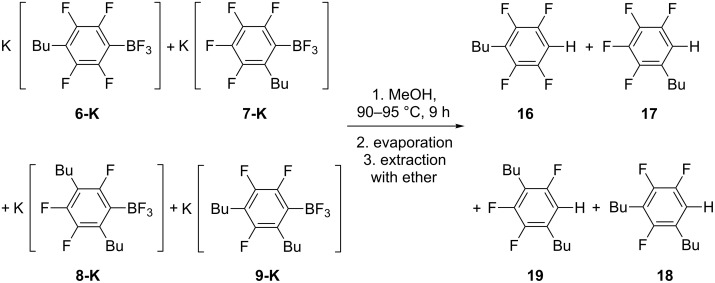
Hydrodeboration of **6-K**, **7-K**, **8-K** and **9-K** in MeOH.

Heating a mixture of **6-K**, **7-K**, **8-K** and **9-K** in MeOH leads to conversion of these salts to **16**, **17**, **18** and **19**, respectively. The molar ratio of the produced polyfluoroarenes is the same as the ratio of their organoboron precursors (Scheme 5).

The ^19^F NMR spectrum of **17** was described [34] and the spectrum of **16** is closely related to the spectrum of known compound **4** [33]. The structures of **18** and **19** are consistent with ^19^F NMR, GC–MS and HRMS data.

**Scheme 6:**
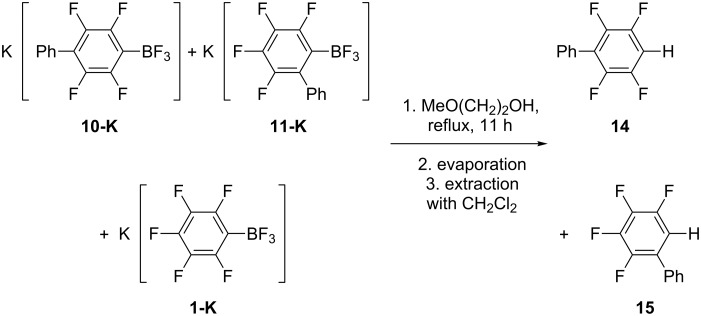
Hydrodeboration of **1-K**, **10-K** and **11-K** in methyl cellosolve.

For characterization of the products derived from **1-K** and PhLi we performed the hydrodeboration of a mixture of **1-K**, **10-K** and **11-K** by stirring it in 2-methoxyethanol under reflux. After evaporation of the alcohol and C_6_F_5_H, the known 2,3,5,6-tetrafluorobiphenyl (**14**) [35–36] and 2,3,4,5-tetrafluorobiphenyl (**15**) [35–36] were obtained (Scheme 6).

**Scheme 7:**
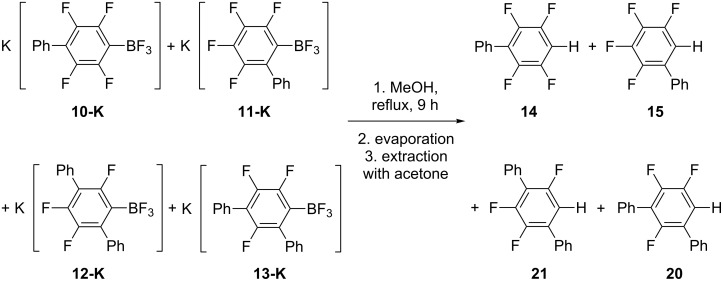
Hydrodeboration of **10-K**, **11-K**, **12-K** and **13-K** in MeOH.

Then a mixture of borates **10-K**, **11-K**, **12-K**, and **13-K** was converted to biphenyls **14**, **15**, and terphenyls **20**, **21**, respectively, and characterized by ^19^F NMR spectroscopy, GC–MS and HRMS (Scheme 7).

## Discussion

**Scheme 8:**
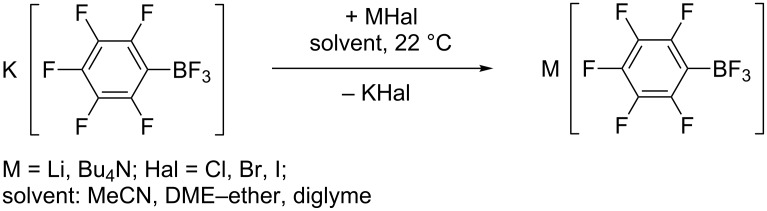
Preparation of **1-Li** and **1-N**.

Above we mentioned that an addition of MeLi or PhLi in ether to a solution of **1-K** in DME caused immediate precipitation. The combination of **1-K** in DME with BuLi in hexanes or PhC≡CLi in ether does not lead to the formation of a solid phase. Because etherial solutions of MeLi and PhLi were prepared from lithium and MeI or PhBr, they contain the corresponding lithium halides. It follows that the precipitate consists of KI and KBr, respectively, and the actual boron-containing reactant is lithium pentafluorophenyltrifluoroborate (**1-Li**). Independently, Li[C_6_F_5_BF_3_] was prepared by metathesis of **1-K** with LiHal (Hal = Cl, Br, I) in an approapriate solvent (Scheme 8). After determination of the salt concentration by ^19^F NMR, **1-Li** was used in DME without isolation. When the metathesis was performed in MeCN, the lithium salt was isolated from MeCN as solid solvate Li[C_6_F_5_BF_3_]·2MeCN. Dissolution of the solvate in DME leads to liberation of free MeCN (^1^H NMR). [Bu_4_N][C_6_F_5_BF_3_] (**1-N**) was prepared in similar way from **1-K** and [Bu_4_N]Br in MeCN and after removal of the solvent from the supernatant it was dissolved in DME or diglyme.

In the course of these experiments we paid attention on distinctions in the NMR spectra of M[C_6_F_5_BF_3_] (M = Li, K, Bu_4_N) (Table 4).

**Table 4:** The ^11^B and ^19^F NMR spectra of M[C_6_F_5_BF_3_]^a^_._

borate	solvent	δ(B)	δ(F)			

			BF_3_	F^2,6^	F^4^	F^3,5^

Li[C_6_F_5_BF_3_]	DME	2.31	−137.2	−134.1	−161.3	−165.9
Li[C_6_F_5_BF_3_]	CD_3_CN	1.73	−135.3	−134.6	−159.9	−164.9
K[C_6_F_5_BF_3_]	DME	2.24	−134.2	−134.4	−161.3	−165.6
K[C_6_F_5_BF_3_] [28]	CD_3_CN	1.81	−133.4	−135.2	−160.7	−165.3
[Bu_4_N][C_6_F_5_BF_3_]	DME	1.89	−132.4	−133.1	−162.6	−166.3
[Bu_4_N][C_6_F_5_BF_3_]	diglyme	1.68	−132.4	−133.1	−162.0	−165.8

^a^in all cases ^1^*J*(B, F) = 43–44 Hz and ^3^*J*(F^4^, F^3,5^) ca. 20 Hz.

The replacement of Li^+^ by K^+^ and Bu_4_N^+^ is accompanied with remarkable changes in the NMR spectra. In the ^11^B NMR spectrum the signal of BF_3_ group shifts from 2.31 (M = Li) to 2.24 (M = K) and 1.89 (M = Bu_4_N) ppm (in DME). In solutions of **1-N** in diglyme and CH_2_Cl_2_ this signal locates at 1.68 and 1.51 ppm, respectively. The opposite is the case in the ^19^F NMR spectra. The signal of BF_3_ group shifts from −137.2 (**1-Li**) to −134.2 (**1-K**) and −132.4 (**1-N**) ppm in DME solution or to −134.1 ppm (**1-N**) in CH_2_Cl_2_. The positions of the fluorine atoms of the C_6_F_5_ moiety are weakly sensitive to the nature of the counteranion although the fluorine atoms F^2,6^ of **1-N** in CH_2_Cl_2_ are somewhat shielded with respect to those in diglyme and DME. It is reasonable to assume that these spectral phenomena reflect the different solvation of M[C_6_F_5_BF_3_]. Detailed investigations in this field are out of the scope of the current research but some qualitative considerations may be outlined. In solution of **1-Li** in DME the lithium cation is strongly coordinated with one or multiple fluorine atoms bonded to boron (“hard”–“hard” interaction) and with solvent molecules to form contact ion pair [37]. The opposite situation is in **1-N** where the bulky tetrabutylammonium cation (“soft”) interacts with those fluorine atom(s) weaker than Li^+^ either in DME and diglyme and this salt forms solvent-separated ion pairs. Potassium pentafluorophenyltrifluoroborate is the intermediate position. The ^11^B and ^19^F NMR chemical shifts of BF_3_ group in **1-K** in DME are closely related to the shifts of **1-Li** in the same solvents and reflect the formation of contact ion pairs. In acetonitrile the salts **1-Li** and **1-N** form solvent-separated ion pairs (^11^B and ^19^F NMR). These observations eludicate the effect of counteractions on the isomer compositions of M[RC_6_F_4_BF_3_]. In DME both salts, **1-Li** and **1-K**, exist as the contact ion pairs and thus the molar ratios of [4-BuC_6_F_4_BF_3_]^−^/[2-BuC_6_F_4_BF_3_]^−^ should be similar. Indeed, the ratio of these products derived from **1-Li** and **1-K** are 1:0.15 and 1:0.18, respectively. Nucleophilic methylation of **1-Li** also results in a related value 1:(0.10–0.13) (Table 1, Table 2), i.e., the isomer ratio remains constant within the experimental error. Salt **1-N** exists in DME as solvent-separated ion pair. Because of this the fluorine atom of BF_3_ is more accessible to coordinate RLi and the ratio [4-BuC_6_F_4_BF_3_]^−^ to [2-BuC_6_F_4_BF_3_]^−^ becomes 1:0.66. Diglyme is a more bulky ligand and that ratio decreases to 1:0.35 (Table 2, entries 5 and 6).

**Scheme 9:**
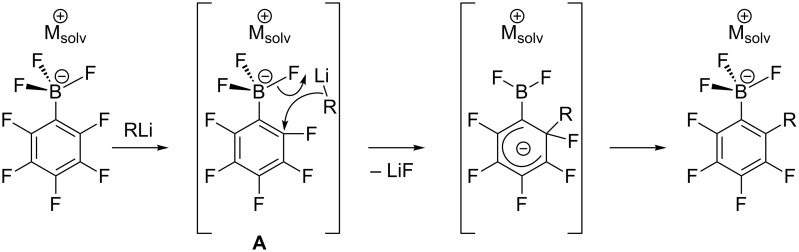
Formation of 2-R-tetrafluorophenyltrifluoroborates.

**Scheme 10:**
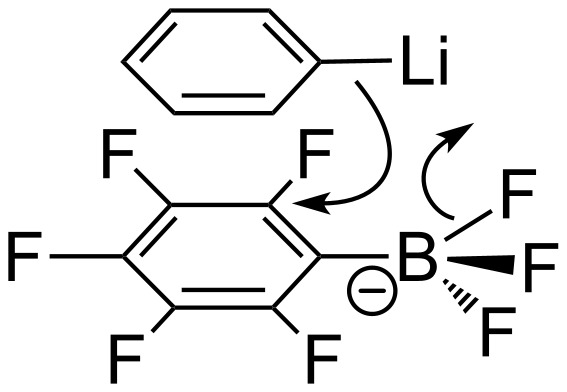
Interaction between C_6_F_5_BF_3_^−^ and PhLi.

When R = Ph, the ratio [4-PhC_6_F_4_BF_3_]^−^/[2-PhC_6_F_4_BF_3_]^−^ derived from **1-Li** and 0.8 equiv of PhLi increases up to 1:(0.9–1.3) (Table 3, entries 2, 3 and 6). Other data from Table 3 are not reliable for comparison because the initial ratio is remarkably corrupted by the further reactions. We believe that the enrichment of the reaction mixture in [2-PhC_6_F_4_BF_3_]^−^ occurs because of an additional stabilization of transition state **A** (Scheme 9) due to the π-stacking interactions between C_6_H_5_ and C_6_F_5_ moieties (Scheme 10), which is excluded in cases of nucleophilic alkylation.

**Scheme 11:**
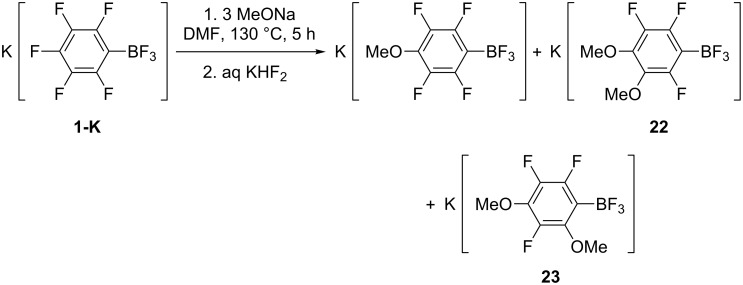
Interaction of **1-K** with MeONa.

While reactions of K[C_6_F_5_BF_3_] with C-nucleophiles give the significant amount of K[2-NuC_6_F_4_BF_3_] the related isomers are not formed under the action of O-nucleophiles, RONa, and N-nucleophiles, AzNa (Az = azol-1-yl). Only a few borates K[3,4-Az_2_C_6_F_3_BF_3_] (Az = indol-1-yl, benzimidazol-1-yl) were detected in the last reaction [32]. The substitution of a fluorine atom in **1-K** by the RO group (in the reaction with 1 equiv of RONa) gives only K[4-ROC_6_F_4_BF_3_] [31]. However, the reaction with 3 equiv of MeONa under the same conditions gives potassium 3,4-dimethoxytrifluorophenyltrifluoroborate (**22**) and potassium 2,4-dimethoxytrifluorophenyltrifluoroborate (**23**) besides K[4-MeOC_6_F_4_BF_3_] and 2,3,5,6-tetrafluorophenol. The latter are formed because of some moisture in MeOH [31] (Scheme 11).

In our opinion, the reason of the negligible content of isomer [2-NuC_6_F_4_BF_3_]^−^ is the lesser affinity to fluoride of Na^+^ and K^+^ compared with Li^+^ (considerations on the relative fluoride affinities are grounded on the crystal lattice energy of LiF (1027 kJ·mol^−1^), NaF (914 kJ·mol^−1^) and KF (812 kJ·mol^−1^) [38]) and the ionic nature of RO–M and RR'N–M (M = K, Na) bonds in the examined nucleophiles. Even in spite of the possible coordination of K^+^ or Na^+^ with the BF_3_ group, free anions RO^−^ or RR'N^−^ attack the carbon atom C-4 rather than C-2 and C-6.

The tolerance of **1-K** towards PhC≡CLi is a consequence of the low nucleophilicity of PhC≡CLi. For instance, C_6_F_6_ and C_6_F_5_C_6_F_5_ do not react with PhC≡CLi in ether, although the addition of a coordinating solvent (DME, diglyme [39], THF [40]) accelerates nucleophilic substitution. C_6_F_5_CH_3_ bearing the non-electron-withdrawing substituent CH_3_ (σ_I_ ≈ 0.0 [41]) remains inert towards PhC≡CLi even in DME–ether [39].

**Scheme 12:**
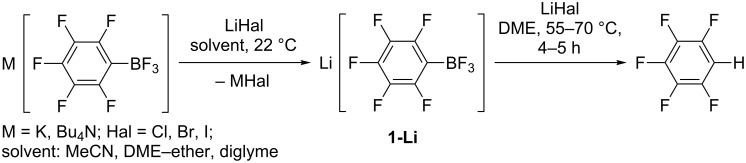
Interaction of M[RC_6_F_5_BF_3_] with lithium halides.

When pentafluorophenyltrifluoroborates react with MeLi (Table 1, entries 5 and 6) or PhLi (Table 3, entry 6) in DME–ether at elevated temperature, partial hydrodeboration of M[RC_6_F_4_BF_3_] as well as M[R_2_C_6_F_3_BF_3_] occurs in addition to nucleophilic substitution. This process was not observed for BuLi in hexanes and PhC≡CLi in ether at 40–60 °C. We assumed that this side reaction proceeds because of the interaction of M[RC_6_F_5_BF_3_] with LiHal, which is present in solutions of MeLi and PhLi in ether and absent in solutions of BuLi and PhC≡CLi. Indeed, heating **1-K** with LiHal (Hal = I, Br, or Cl) in DME at 55–70 °C leads to the formation of C_6_F_5_H. [Bu_4_N][C_6_F_5_BF_3_] reacts with LiI in diglyme in a similar way. Because the cations K^+^ or Bu_4_N^+^ are replaced with Li^+^ in all cases, the reactions proceed via the lithium salt. Actually, the salt Li[C_6_F_5_BF_3_] prepared from **1-K** and an excess of LiI at 22 °C in quantitative yield converted to C_6_F_5_H in high yield when being heated in DME at 55–70 °C (Scheme 12). A similar reaction of **1-K** occurs in MeOH in the presence of LiCl [33].

**Scheme 13:**
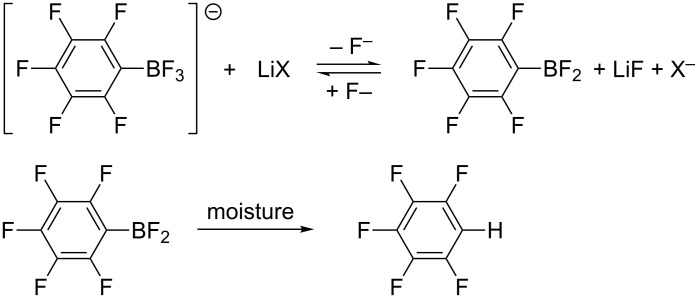
Assumed role of lithium halides.

Presumably, one role of lithium halides is the fluoride abstraction from lithium aryltrifluoroborate (or significant polarization of the B–F bond) and the subsequent hydrodeboration of aryldifluoroborane by residual moisture in the solvent (Scheme 13). This assumption is evidenced by the fact that lithium salts are used as catalysts for the reverse transformation of organotrifluoroborates in the corresponding organoboronic acids.

In the absence of LiHal, borate **1-Li** does not change in DME (22 °C, 1 week; 60–68 °C, 5 h), and neither does **1-N** in diglyme (22 °C, 1 year; 60–68 °C, 5 h). This observation contrasts with the reactivity of the close analogue, Li[C_6_F_5_B(OMe)_3_], which undergoes hydrodeboration in methanol, acetone or acetonitrile. Li[C_6_F_5_B(OMe)_3_] converts to Li[(C_6_F_5_)_2_B(OMe)_2_] and Li[B(OMe)_4_] at 22 °C in DME. Also disproportionation of M[C_6_F_5_B(OMe)_3_] (M = Li, K) proceeds in weakly coordinated CH_2_Cl_2_ in the presence of [Bu_4_N]Br or KF while in the absence of other salts the lithium salt is relatively stable. This phenomenon was explained by the formation of a dinuclear methoxy-bridged borate intermediate [C_6_F_5_B(OMe)_2_–(μ-OMe)–B(OMe)_2_C_6_F_5_]^−^ (**B**) followed the migration of both the aryl and the methoxy groups. If Li^+^ and [C_6_F_5_B(OMe)_3_]^−^ form a contact ion pair (solution of Li[C_6_F_5_B(OMe)_3_] in CH_2_Cl_2_), such migration of ^−^OMe and its subsequent elimination is hindered [42]. In the case of pentafluorophenyltrifluoroborates the similar conversion does not occur even with Li[C_6_F_5_BF_3_] in DME (contact ion pairs) due to the higher Lewis acidity of C_6_F_5_BF_2_ relative to C_6_F_5_B(OMe)_2_, which prevents the formation of fluoro-bridged intermediates such as **B**.

## Conclusion

1. Nucleophilic substitution of fluorine atoms in M[C_6_F_5_BF_3_] (M = K, Li, Bu_4_N) with MeLi or BuLi at 22 °C and subsequent treatment with aqueous KF leads preferentially to K[4-RC_6_F_4_BF_3_] while K[2-RC_6_F_4_BF_3_] is a minor isomer (R = Me, Bu). Under the same conditions, the reaction with PhLi gives approximately equimolar amounts of K[4-PhC_6_F_4_BF_3_] and K[2-PhC_6_F_4_BF_3_] and remarkable amounts of K[2,5-Ph_2_C_6_F_3_BF_3_] and K[2,4-Ph_2_C_6_F_3_BF_3_]. The substitution of two fluorine atoms by the butyl group at 55–60 °C gives the related isomers while a complex mixture forms from K[C_6_F_5_BF_3_] and MeLi at the same temperature. K[C_6_F_5_BF_3_] does not react with PhC≡CLi in DME–ether under reflux because the low reactivity of C-nucleophile.

2. Because solutions of MeLi and PhLi contain LiBr or LiI, the salts M[C_6_F_5_BF_3_] (M = K, Bu_4_N) undergo metathesis with the formation of Li[C_6_F_5_BF_3_]. The latter is the actual reagent in the reactions of nucleophilic substitution. BuLi in hexanes does not contain LiHal and thus it reacts with K[C_6_F_5_BF_3_].

3. According to the ^11^B and ^19^F NMR data, salts Li[C_6_F_5_BF_3_] and K[C_6_F_5_BF_3_] exists as contact ion pairs in DME and solvent-separated ion pairs in CH_3_CN. [Bu_4_N][C_6_F_5_BF_3_] forms solvent-separated ion pairs in DME or diglyme. The sort of solvation affects the ratio M[4-RC_6_F_4_BF_3_]/M[2-RC_6_F_4_BF_3_]: In case of the contact ion pairs the contribution of the ortho alkylation is minimal (M = K, Li). During nucleophilic phenylation the π–stacking interaction between C_6_H_5_ and C_6_F_5_ moieties can be responsible for increased yield of M[2-PhC_6_F_4_BF_3_].

4. The formation of M[2-RC_6_F_4_BF_3_] proceeds through the coordination of RLi (polarized C–Li bond) to a fluorine atom of the BF_3_ moiety and subsequent elimination of LiF. In contrary, the cation–anion bonds in O-nucleophiles and in N-nucleophiles are ionic (M = K, Na) and the fluoride affinities of K^+^ and Na^+^ are smaller than that of Li^+^. These factors determine the reaction route with K[C_6_F_5_BF_3_] by a simple S_N_2 mechanism.

## Supporting Information

File 1 Full experimental details and compounds characterization data.
