# Supplementary material for: Substitution of fluorine in M[C6F5BF3] with organolithium compounds: distinctions between O- and N-nucleophiles
Source: Beilstein J Org Chem. 2017 Apr 12;13:703–13. doi: 10.3762/bjoc.13.69 (PMC5405681; doi:10.3762/bjoc.13.69)
Supplement: File 1 — Full experimental details and compounds characterization data. [file Beilstein_J_Org_Chem-13-703-s001.pdf]

Supporting information

for

Substitution of fluorine in M[$C_6F_5BF_3$] with organolithium compounds: distinctions between O- and N-nucleophiles

Anton Yu. Shabalin[1,2], Nicolay Yu. Adonin*[1,2] and Vadim V. Bardin[2,3]

Address: [1]G.K. Boreskov Institute of Catalysis, SB RAS, Acad. Lavrentjev Ave. 5, Novosibirsk, 630090, Russian Federation, [2]Novosibirsk State University, Pirogova str., 2, Novosibirsk, 630090, Russian Federation and [3]N.N. Vorozhtsov Novosibirsk Institute of Organic Chemistry, SB RAS, Acad. Lavrentjev Ave. 9, Novosibirsk, 630090, Russian Federation

Email: Nicolay Yu. Adonin* - adonin@catalysis.ru

*Corresponding author

Full experimental details and compounds characterization data

Table of Contents

1. General 2

2. Preparation of [Bu_4N][$C_6F_5BF_3$] (1-N) 2

3. Reaction of K[$C_6F_5BF_3$] with methyllithium 2

4. Reaction of M[$C_6F_5BF_3$] with butyllithium 4

5. Reaction of K[$C_6F_5BF_3$] with phenyllithium 6

6. Hydrodeboration of potassium polyfluoroaryltrifluoroborates with MeOH 7

7. Attempted reaction of K[$C_6F_5BF_3$] with phenylethynyllithium 9

8. Reaction of M[$C_6F_5BF_3$] with alkali halides 10

9. Stability of M[$C_6F_5BF_3$] in solution 11

10. Reaction of K[$C_6F_5BF_3$] with MeONa (excess) 11

1. General

The NMR spectra were recorded on Bruker AVANCE 300 (^{1}H at 300.13 MHz, ^{19}F at 282.40 MHz), and Bruker AVANCE 600 (^{11}B at 192.60 MHz) spectrometers. The chemical shifts are referenced to TMS (^{1}H), CCl_3F (^{19}F, with C_6F_6 as secondary reference (−162.9 ppm)), and $BF_3{\cdot}OEt_2/CDCl_3$ (15% v/v) (^{11}B), respectively. GC–MS analysis was done on a Hewlett-Packard 1800A (with HP-5MS column) instrument. High-resolution mass spectra were recorded on a Thermo Scientific DFS spectrometer in EI mode (70 eV). Diglyme (Acros) and 1,2-dimethoxyethane (Acros) were stirred with CaH_2, distilled and stored over molecular sieves 4Å under an argon atmosphere. NaH (60% dispersion in oil) (Sigma-Aldrich), BuLi in hexanes (Acros) was used as supplied. Ether was passed through column with alumina dried at 500 °C for 4 h and stored over sodium. Potassium pentafluorophenyltrifluoroborate [1], MeLi in ether [2], PhLi in ether [3], and PhC≡CLi in ether [4] were prepared as described and stored at 5–8 °C. The actual concentration of organolithium was controlled before use by titration with 0.1 N HCl (MeLi and PhC≡CLi) and by Watson's method [5] (PhLi and BuLi). All manipulations with organolithium reagents were performed under an atmosphere of dry argon.

The solubility of $K[C_6F_5BF_3]$ in DME (22 °C) exceeds 225 mg (0.82 mmol) per mL. The solubility of $[Bu_4N]I$ in diglyme (22 °C) is 3.4 mg (0.008 mmol) per mL.

2. Preparation of $[Bu_4N][C_6F_5BF_3]$ (1-N)

Suspension of $K[C_6F_5BF_3]$ (125 mg, 0.45 mmol) and $[Bu_4N]Br$ (151 mg, 0.47 mmol) in MeCN (3 mL) was stirred at 22 °C for 2 h and centrifuged. The supernatant was evaporated to dryness under reduced pressure to yield $[Bu_4N][C_6F_5BF_3]$ (colorless oil, 200 mg, 93%).

$[Bu_4N][C_6F_5BF_3]$ (1-N). ^{11}B NMR (CD_2Cl_2): δ 1.51 (q, ^{1}J(B, F) = 44 Hz, BF_3). ^{19}F NMR (CD_2Cl_2): δ –134.1 (q (1:1:1:1), ^{1}J(F, B) = 44 Hz, 3F, BF_3), –135.4 (ddq (1:1:1:1), $^{3}J(F^2, F^3)$ = 24 Hz, $^{5}J(F^2, F^5)$ = 12 Hz, $^{4}J(F^2, BF)$ = 12 Hz, 2F, $F^{2,6}$), –161.7 (t, $^{3}J(F^4, F^{3,5})$ = 20 Hz, 1F, F^4), –166.2 (m, 2F, $F^{3,5}$).

3. Reaction of $K[C_6F_5BF_3]$ with methyllithium

3.1. A three-necked flask equipped with a reflux condenser topped with gas inlet/outlet tube and bubbler, a Teflon-coated magnetic bar, septum inlet, and adapter

for an addition of MeLi was filled with dry argon and charged with K[$C_6F_5BF_3$] and DME. The colorless solution was stirred for 10-15 min. MeLi in ether was siphoned into a measuring vessel and then added in portions to the above solution. Immediately white precipitate formed. At the end of the reaction, a saturated aqueous solution of KF (10 mL) was added to the suspension, and after 1 h an organic phase was decanted, dried with $MgSO_4$ and analyzed by the ^{19}F NMR spectroscopy (Table 1).

3.2. K[$C_6F_5BF_3$] (276 mg, 1.0 mmol) in DME (10 mL) was reacted with 0.36 M MeLi in ether (10 mL, 3.6 mmol) at 22 °C for 3 h, and treated with saturated aqueous solution of KF (20 mL). The organic phase contained **2-K** (0.55 mmol) and **3-K** (0.05 mmol) (Table 1, entry 4). Solution was evaporated to dryness on an evaporator, solid residue was washed with benzene (10 mL) and dried at 40 °C under reduced pressure to yield **2-K** and **3-K** (1:0.10) (206 mg).

K[4-Me$C_6F_4BF_3$] (2-K). ^{1}H NMR (acetone-d$_6$): δ 2.15 (t, ^{4}J(H$_3$C, F$^{3,\ 5}$) = 2 Hz, 3H, CH$_3$). ^{11}B NMR and ^{19}F NMR spectra coincided with reported ones [6].

K[2-Me$C_6F_4BF_3$] (3-K). ^{1}H NMR (acetone-d$_6$): δ 2.28 (m, 3H, CH$_3$). ^{11}B NMR (acetone-d$_6$): δ 2.45 (q, ^{1}J(B, F) = 48 Hz, BF$_3$). ^{19}F NMR (acetone-d$_6$): δ –132.7 (dq (1:1:1:1), ^{4}J(F^6, B*F*) = 15 Hz, ^{1}J(F, B) = 48 Hz, 3F, BF$_3$), –135.0 (m, 1F, F^6), –145.3 (dd, ^{5}J(F^3, F^6) = 13 Hz, ^{3}J(F^3, F^4) = 21 Hz, 1F, F^3), –163.9 (t, ^{3}J(F^5, F^{4,6}) = 20 Hz, 1F, F^5), –164.2 (t, ^{3}J(F^4, F^{3,5}) = 20 Hz, 1F, F^4).
Anal. calcd for $C_7H_3BF_7K$ (270.00): C 31.1; H 1.12; F 49.26; found: C 30.6; H 1.11; F 48.9.

3.3. K[$C_6F_5BF_3$] (128 mg, 0.46 mmol) in DME (5 mL) was reacted with 0.92 M MeLi in ether (2 mL, 1.8 mmol) at 60–62 °C for 1 h. Suspension was cooled to 25 °C. The supernatant contained 4 (0.02 mmol), and several unknown compounds. After stirring of suspension with saturated aqueous solution of KF (10 mL) for 8 h the quantity of **4** increased to 0.14 mmol and resonances of pentafluorobenzene (0.01 mmol) and 2,3,4,5-tetrafluorotoluene (0.01 mmol) appeared besides unknown products (^{19}F NMR).

4. Reaction of M[$C_6F_5BF_3$] with butyllithium

4.1. Reaction of K[$C_6F_5BF_3$] (94 mg, 0.34 mmol) in DME (4 mL) with BuLi in hexanes (2.4 M, 0.3 mL, 0.72 mmol) was performed at 22 °C over a period of 2 h as described in 3.1. The ^{19}F NMR spectrum showed signals of K[4-Bu$C_6F_4BF_3$] (0.22 mmol), and K[2-Bu$C_6F_4BF_3$] (0.04 mmol) (Table 2, entry 1).

4.2. BuLi (2.4 M, 1.2 mL, 2.8 mmol) in hexanes was added in portions to stirred solution of K[$C_6F_5BF_3$] (279 mg, 1.0 mmol) in DME (10 mL) and maintained at 25 °C for 2 h. After treatment with aqueous KF for 1 h, organic phase was decanted and dried with $MgSO_4$. The ^{19}F NMR spectroscopy showed the presence of **6-K** (0.65 mmol) and **7-K** (0.07 mmol) and absence of **1-K** (Table 2, entry 2).
Solutions of **6-K** and **7-K** from several experiments were combined and evaporated to dryness under reduced pressure. Solid residue was washed with CH_2Cl_2 (3 mL), suspension was centrifugated, organic phase was decanted and solid was dried on an air. An isomer mixture K[4-Bu$C_6F_4BF_3$] (0.49 mmol) and K[6-Bu$C_6F_4BF_3$] (0.10 mmol) (185 mg) was obtained (^{19}F NMR).

K[4-Bu$C_6F_4BF_3$] (6-K). ^{1}H NMR (acetone-d$_6$): δ 2.66 (t, ^3J (H^1, H^2) = 7.5 Hz, 2H, H^1), 1.54 (tt, ^3J(H^2, H^1) = 7.5 Hz, ^3J(H^2, H^3) = 7.2 Hz, 2H, H^2), 1.34 (qt, ^3J(H^3, H^4) = 7.2 Hz, ^3J(H^3, H^2) = 7.4 Hz, 2H, H^3), 0.91 (t, ^3J(H^4, H^3) = 7.4 Hz, 3H, H^4). ^{11}B NMR (acetone-d$_6$): δ 1.93 (q, ^1J(B, F) = 44 Hz, BF_3). ^{19}F NMR (acetone-d$_6$): δ –133.9 (q (1:1:1:1), ^1J(F, B) = 43 Hz, 3F, BF_3), –135.7 (ddq (1:1:1:1), ^3J(F^2, F^3) = 24 Hz, ^5J(F^2, F^5) = 12 Hz, ^4J(F^2, B*F*) = 12 Hz, 2F, F^{2,6}), –148.2 (dd, ^3J(F^3, F^2) = 24 Hz, ^5J(F^3, F^6) = 15 Hz, 2F, F^{3,5}).

K[2-Bu$C_6F_4BF_3$] (7-K). ^{1}H NMR (acetone-d$_6$): δ 2.66 (t, ^3J (H^1, H^2) = 7.5 Hz, 2H, H^1), 1.54 (tt, ^3J(H^2, H^1) = 7.5 Hz, ^3J(H^2, H^3) = 7.2 Hz, 2H, H^2), 1.34 (qt, ^3J(H^3, H^4) = 7.2 Hz, ^3J(H^3, H^2) = 7.4 Hz, 2H, H^3), 0.91 (t, ^3J(H^4, H^3) = 7.4 Hz, 3H, H^4). ^{11}B NMR (acetone-d$_6$): δ 2.50 (q, ^1J(B, F) = 48 Hz, BF_3). ^{19}F NMR (acetone-d$_6$): δ –132.4 (q (1:1:1:1), ^1J(F, B) = 44 Hz, 3F, BF_3), –134.7 (m, 1F, F^6), –146.8 (dd, ^3J(F^3, F^4) = 20 Hz, ^5J(F^3, F^6) = 14 Hz, 1F, F^3), –163.7 (t, ^3J(F^5, F^{4,6}) = 20 Hz, 1F, F^5), –163.9 (t, ^3J(F^4, F^{3,5}) = 20 Hz, 1F, F^4).
Anal. calcd for $C_{10}H_9BF_7K$ (312.08): C 38.5; H 2.91; B 3.46; F 42.61; found: C 38.5; H 3.11; B 3.55; F 42.6.

4.3. BuLi (2.4 M, 0.5 mL, 1.2 mmol) in hexanes was added in portions to stirred solution of K[$C_6F_5BF_3$] (162 mg, 0.59 mmol) in DME (3 mL) and maintained at 55–60 °C for 1 h. The ^{19}F NMR spectroscopy showed the presence of **6-K** (0.42 mmol), **7-K** (0.03 mmol), and presumably, **8** (0.02 mmol) and **9** (0.03 mmol) (Table 2, entry 3).

K[2,5-$Bu_2C_6F_3BF_3$] (8). ^{19}F NMR (DME): δ –116.3 (dq (1:1:1:1), ^5J(F^6, F^3) = 15 Hz, ^4J(F^6, B*F*) = 15 Hz, 1F, F^6), –132.9 (br, 3F, BF_3), –145.7 (d, ^3J(F^4, F^3) = 21 Hz, 1F, F^4), –148.3 (m, 1F, F^3).

K[2,4-$Bu_2C_6F_3BF_3$] (9). ^{19}F NMR (DME): δ –128.0 (d, ^5J(F^3, F^6) = 17 Hz, 1F, F^3), –132.9 (br q, 3F, BF_3), –137.0 (m, 1F, F^6), –144.5 (d, ^3J(F^5, F^6) = 23 Hz, 1F, F^5).

4.4. Reaction of K[$C_6F_5BF_3$] (87 mg, 0.32 mmol) in DME (2 mL) with BuLi in hexanes (2.4 M, 0.3 mL, 0.72 mmol) was performed in similar way (22 °C, 1 h). After addition of KF·2H_2O (50 mg) suspension was stirred for an additional 2 h and filtered. Filtrate was evaporated to dryness on evaporator to form white solid (88 mg). Crystallization from MeCN gave analytically pure K[4-$BuC_6F_4BF_3$].

K[4-$BuC_6F_4BF_3$] (6-K). ^{1}H NMR (CD_3CN): *δ* 2.66 (t, ^3J (H^1, H^2) = 7.5 Hz, 2H, H^1), 1.54 (tt, ^3J(H^2, H^1) = 7.5 Hz, ^3J(H^2, H^3) = 7.7 Hz, 2H, H^2), 1.34 (qt, ^3J(H^3, H^4) = 7.4 Hz, ^3J(H^3, H^2) = 7.3 Hz, 2H, H^3), 0.91 (t, ^3J(H^4, H^3) = 7.3 Hz, 3H, H^4). ^{19}F NMR (CD_3CN): *δ* –133.6 (q (1:1:1:1), ^1J(F, B) = 43 Hz, 3F, BF_3), –136.7 (ddq (1:1:1:1), ^3J(F^2, F^3) = 23 Hz, ^5J(F^2, F^5) = 15 Hz, ^4J(F^2, B*F*) = 12 Hz, 2F, F^{2,6}), –147.9 (dd, ^3J(F^3, F^2) = 23 Hz, ^5J(F^3, F^6) = 15 Hz, 2F, F^{3,5}).
Anal. calcd for $C_{10}H_9BF_7K$ (312.08): C 38.49; H 2.91; B 3.46; F 42.61; found: C 39.0; H 2.15; B 3.46; F 42.8.

4.5. Anhydrous LiCl (68 mg, 1.6 mmol) was added to a stirred solution of K[$C_6F_5BF_3$] (418 mg, 1.50 mmol) in DME (6 mL) and formed suspension was stirred at 25 °C for 1 h. BuLi (2.4 M, 1.3 mL, 3.1 mmol) in hexanes was injected with syringe in portions and stirring was continued for an additional 2 h. After treatment with aqueous KF (10 mL) for 1 h, organic phase was decanted and dried with $MgSO_4$. The ^{19}F NMR spectroscopy showed the presence of **6-K** (1.00 mmol), **7-K** (0.15 mmol) and 2,3,5,6-

C_6F_4HBu (0.10 mmol) besides **1-K** (0.04 mmol), and 2,3,4,5-C_6F_4HBu (0.04 mmol) (Table 2, entry 4).

4.6. Solution of $[Bu_4N][C_6F_5BF_3]$ (0.90 mmol) in diglyme (6 mL) was cooled to 5 °C and BuLi in hexanes (2.5 M, 1 mL, 2.5 mmol) was added with syringe to keep temperature below 8 °C. Formed suspension was stirred at 22 °C for 2 h and acidified with HOAc (0.2 mL). Solution contained **1-N** (0.19 mmol), **6-N** (0.54 mmol), and **7-N** (0.19 mmol) (^{19}F NMR) (Table 2, entry 5).

5. Reaction of $K[C_6F_5BF_3]$ with phenyllithium

5.1. A three-necked flask equipped with a reflux condenser topped with bubbler and gas inlet/outlet tube, a Teflon-coated magnetic bar, septum inlet, and adapter for an addition of PhLi was filled with dry argon and charged with $K[C_6F_5BF_3]$ and DME. The colorless solution was stirred for 10–15 min. PhLi in ether was siphoned into a measuring vessel and then added in portions to above solution. Immediately white precipitate formed. At the end saturated aqueous solution of KF (10 mL) was added to suspension, and after 1 h an organic phase was decanted and analyzed by ^{19}F NMR spectroscopy (Table 3).

$K[4\text{-}PhC_6F_4BF_3]$ (10-K). ^{19}F NMR (DME – ether): δ –134.0 (br. q (1:1:1:1), 3F, BF_3), –135.2 (m, 2F, $F^{2,6}$), –147.3 (dd, $^3J(F^3, F^2)$ = 24 Hz, $^5J(F^3, F^6)$ = 14 Hz, 2F, $F^{3,5}$).

$K[2\text{-}PhC_6F_4BF_3]$ (11-K). ^{19}F NMR (DME – ether): δ –130.9 (br. q (1:1:1:1), 3F, BF_3), –134.5 (m, 1F, F^6), –142.8 (dd, $^3J(F^3, F^4)$ = 24 Hz, $^5J(F^3, F^6)$ = 14 Hz, 1F, F^3), –161.4 (dd, $^3J(F^4, F^3)$ = 24 Hz, $^3J(F^4, F^5)$ = 20 Hz, 1F, F^4), –162.7 (dd, $^3J(F^5, F^6)$ = 20 Hz, $^3J(F^5, F^4)$ = 20 Hz, 1F, F^5).

$K[2,5\text{-}Ph_2C_6F_3BF_3]$ (12). ^{19}F NMR (DME – ether): δ –131.0 (br, 4F, 1F^6 and BF_3), –143.3 (dd, $^3J(F^4, F^3)$ = 23 Hz, $^4J(F^4, F^6)$ = 4 Hz, 1F, F^4), –146.3 (dd, $^3J(F^3, F^4)$ = 23 Hz, $^5J(F^3, F^6)$ = 16 Hz, 1F, F^3).

$K[2,4\text{-}Ph_2C_6F_3BF_3]$ (13). ^{19}F NMR (DME – ether): δ –121.5 (d, $^5J(F^3, F^6)$ = 17 Hz, 1F, F^3), –131.0 (br, 3F, BF_3), –137.5 (ddq (1:1:1:1), $^3J(F^6, F^5)$ = 23 Hz, $^5J(F^6, F^3)$ = 17 Hz, $^4J(F^6, BF)$ = 13 Hz, 1F, F^6), –142.5 (d, $^3J(F^5, F^6)$ = 23 Hz, 1F, F^6).

5.2. A four-necked flask equipped with a reflux condenser topped with bubbler and gas inlet/outlet tube, a Teflon-coated magnetic bar, thermometer, septum inlet, and adapter for addition of PhLi was filled with dry argon and charged with $K[C_6F_5BF_3]$ (132 mg, 0.48 mmol) in DME (5 mL). PhLi in ether (2.2 mL, 0.77 mmol) was added in portions. Immediately white suspension formed. The reaction mixture was stirred at 37–40 °C for 1 h, cooled to 22 °C and filtered. The filtrate contained **1-Li** (0.02 mmol), **10-Li** (0.16 mmol, **11-Li** (0.15 mmol), **9-Li** (0.02 mmol), 2,3,5,6-C_6F_4HPh (0.02 mmol) and 2,3,4,5-C_6F_4HPh (0.01 mmol) (Table 3, entry 6).

6. Hydrodeboration of potassium polyfluoroaryltrifluoroborates with MeOH

6.1. An ampoule was equipped with a magnetic bar, charged with $K[BuC_6F_4BF_3]$, $K[Bu_2C_6F_3BF_3]$ (see 4.3.) and MeOH (5 mL) and flame-sealed. The suspension was stirred at 90–100 °C (bath) for 9 h. After cooling, volatiles were evaporated under reduced pressure and residue was extracted with ether (2 mL). The extract contained **16**, **17**, **18**, **19** and 1,5-dibutyl-2,3,4-trifluorobenzene in molar ratio 11:10:18:10:3 (^{19}F NMR).

1-Butyl-2,3,5,6-tetrafluorobenzene (16). ^{19}F NMR (ether): δ –140.5 (ddd, $^3J(F^3, H^4)$ = 10 Hz, $^3J(F^3, F^2)$ = 22 Hz, $^5J(F^3, F^6)$ = 13 Hz, 2F, $F^{3, 5}$), –145.2 (ddd, $^4J(F^2, H^4)$ = 10 Hz, $^3J(F^2, F^3)$ = 22 Hz, $^5J(F^2, F^5)$ = 13 Hz, 2F, $F^{2, 6}$).

1-Butyl-2,3,4,5-tetrafluorobenzene (17). ^{19}F NMR (ether): δ –141.8 (m, 1F, F^5), –144.4 (dd, $^3J(F^2, F^3)$ = 20 Hz, $^5J(F^2, F^5)$ = 14 Hz, 1F, F^2), –157.9 (dddd, $^3J(F^3, F^2)$ = 19 Hz, $^3J(F^3, F^4)$ = 20 Hz, $^4J(F^3, F^5)$ = 5 Hz, $^5J(F^3, H^6)$ = 5 Hz, 1F, F^3), –161.4 (dd, $^3J(F^4, F^3)$ = 20 Hz, $^4J(F^4, F^5)$ = 22 Hz, 1F, F^4) (lit. δ –141.3, –145.3 , –157.2 and –160.4 (dt, 1F) [7]).

1,3-Dibutyl-2,4,5-trifluorobenzene (18). ^{19}F NMR (ether): δ –126.2 (d, $^5J(F^2, F^5)$ = 17 Hz, 1F, F^2), –139.2 (m, 1F, F^5), –144.1 (d, $^3J(F^4, F^5)$ = 23 Hz, 1F, F^4).

1,4-Dibutyl-2,3,5-trifluorobenzene (19). ^{19}F NMR (ether): δ –114.2 (dd, $^3J(F^5, H^6)$ = 9 Hz, $^5J(F^5, F^2)$ = 16 Hz, 1F, F^5), –140.6 (dd, $^3J(F^3, F^2)$ = 21 Hz, $^5J(F^3, H^6)$ = 5 Hz, 1F, F^3), –149.1 (dd, $^3J(F^2, F^3)$ = 21 Hz, $^5J(F^2, F^5)$ = 15 Hz, 1F, F^2).

1,5-Dibutyl-2,3,4-trifluorobenzene. ^{19}F NMR (ether): δ –144.0 (d, ^{3}J(F^{2}, F^{3}) = 22 Hz, 2F, F^{2,4}), –161.0 (t, ^{3}J(F^{3}, F^{2,4}) = 22 Hz, 1F, F^{3}) (tentative assignment).
GC-MS. M^{+} (%): 206 (2%), 206 (17%), 206 (12%) ($C_{10}H_{10}F_4$), 244 (2%), 244 (6%), 244 (19%), 244 (16%) ($C_{14}H_{19}F_3$).
HRMS (ESI) (mixture of $C_6HF_4C_4H_9$ and $C_6HF_3(C_4H_9)_2$), *m/z*: calcd. for $C_{10}H_{10}F_4$ 206.0713; found 206.0706; calcd. for $C_{14}H_{19}F_3$ 244.1433; found 244.1428.

6.2. Solution of **1-K**, **10-K** and **11-K** (Table 3, entry 2) was diluted with $MeOCH_2CH_2OH$ (2.5 mL) and refluxed (124 °C, bath) with stirring for 7 h. The solution contained **1-K**, **10-K** (5:1), **14**, **15**, C_6F_5H, K[BF_4] and K[$BF_3OCH_2CH_2OMe$] (^{19}F NMR). The solution was stirred under reflux for an additional 4 h, evaporated under reduced pressure and residue was extracted with CH_2Cl_2 (4 mL). Solution of **14** and **15** was obtained (^{19}F NMR).

2,3,5,6-Tetrafluorobiphenyl (14). ^{19}F NMR (CH_2Cl_2): δ –140.2 (ddd, ^{3}J(F^{3}, H^{4}) = 10 Hz, ^{3}J(F^{3}, F^{2}) = 23 Hz, ^{5}J(F^{3}, F^{6}) = 12 Hz, 2F, F^{3,5}), –144.7 (m, F^{2,6}) (lit. δ –139.9 and –144.3 [8], –139.1 and –143.9 [9]).

2,3,4,5-Tetrafluorobiphenyl (15). ^{19}F NMR (CH_2Cl_2): δ –140.7 (ddd, ^{3}J(F^{5}, F^{4}) = 22 Hz, ^{3}J(F^{5}, H^{6}) = 10 Hz, ^{5}J(F^{5}, F^{2}) = 11 Hz, 1F, F^{5}), –144.9 (ddd, ^{3}J(F^{2}, F^{3}) = 22 Hz, ^{4}J(F^{2}, H^{6}) = 8 Hz, ^{5}J(F^{2}, F^{5}) = 13 Hz, 1F, F^{2}), –156.6 (dd, ^{3}J(F^{3}, F^{4}) = 20 Hz, ^{3}J(F^{3}, F^{2}) = 22 Hz, 1F, F^{3}), –158.5 (ddd, ^{3}J(F^{4}, F^{3}) = 20 Hz, ^{3}J(F^{4}, F^{5}) = 22 Hz, ^{4}J(F^{4}, H^{6}) = 8 Hz, 1F, F^{4}) (lit. δ –140.4, –144.3, –156.0, and 158.0 [8], –139.6 (m, 1F), –143.7 (m, 1F), –155.2 (m, 1F), –157.1 (m, 1F) [9]).

6.3. Solution of borates (Table 3, entries 1 and 3) was evaporated to dryness under reduced pressure and formed solid was stirred with MeOH (6 mL) at 90–95 °C for 6 h in a sealed tube. After evaporation of the solvent, residue was extracted with acetone (2 mL). The extract contained **14**, **15**, **20** and **21** in molar ratio 160:100:15:18 (^{19}F NMR).

2,3,5,6-Tetrafluorobiphenyl (14). ^{19}F NMR (acetone): δ –139.3 (ddd, ^{3}J(F^{3}, H^{4}) = 10 Hz, ^{3}J(F^{3}, F^{2}) = 22 Hz, ^{5}J(F^{3}, F^{6}) = 13 Hz, 2F, F^{3,5}), –143.8 (ddd, ^{3}J(F^{2}, F^{3}) = 22 Hz, ^{4}J(F^{2}, H^{4}) = 8 Hz, ^{5}J(F^{2}, F^{5}) = 13 Hz, 2F, F^{2,6}).

2,3,4,5-Tetrafluorobiphenyl (15). ^{19}F NMR (acetone): δ –139.8 (ddd, ^3J(F^5, F^4) = 21 Hz, ^3J(F^5, H^6) = 11 Hz, ^5J(F^5, F^2) = 12 Hz, 1F, F^5), –142.1 (ddd, ^3J(F^2, F^3) = 22 Hz, ^4J(F^2, H^6) = 10 Hz, ^5J(F^2, F^5) = 15 Hz, 1F, F^2), –156.2 (dddd, ^3J(F^3, F^4) = 20 Hz, ^3J(F^3, F^2) = 22 Hz, ^4J(F^3, F^5) = 2 Hz, ^5J(F^3, H^6) = 2 Hz, 1F, F^3), –158.2 (dddd, ^3J(F^4, F^3) = 20 Hz, ^3J(F^3, F^5) = 21 Hz, ^4J(F^4, F^2) = 2 Hz, ^4J(F^4, H^6) = 8 Hz, 1F, F^4).

1,3-Diphenyl-2,4,5-trifluorobenzene (20). ^{19}F NMR (MeOH): δ –122.7 (d, ^5J(F^2, F^5) = 15 Hz, 1F, F^2), –139.4 (ddd, ^3J(F^4, F^5) = 22 Hz, ^4J(F^4, H^6) = 9 Hz, ^4J(F^4, F^2) = 4 Hz, 1F, F^4), –142.1 (ddd, ^3J(F^5, F^4) = 22 Hz, ^3J(F^5, H^6) = 11 Hz, ^5J(F^5, F^2) = 15 Hz, 1F, F^5). ^{19}F NMR (acetone): δ –121.9 (d, ^5J(F^2, F^5) = 16 Hz, 1F, F^2), –138.8 (d, ^3J(F^4, F^5) = 22 Hz, 1F, F^4), –139.2 (dd, ^3J(F^5, F^4) = 22 Hz, ^3J(F^5, H^6) = 8 Hz, 1F, F^5; ^5J(F^5, F^2) value was not determined because overlappinig with signal F^4).

1,4-Diphenyl-2,3,5-trifluorobenzene (21). ^{19}F NMR (MeOH): δ –119.5 (dd, ^3J(F^5, H^6) = 10 Hz, ^5J(F^5, F^2) = 14 Hz, 1F, F^5), –137.9 (d, ^3J(F^3, F^5) = 21 Hz, 1F, F^3), –147.4 (ddd, ^3J(F^2, F^3) = 21 Hz, ^4J(F^2, H^6) = 5 Hz, ^5J(F^2, F^5) = 14 Hz, 1F, F^2). ^{19}F NMR (acetone): δ –119.5 (ddd, ^3J(F^5, H^6) = 11 Hz, ^4J(F^5, F^3) = 4 Hz, ^5J(F^5, F^2) = 15 Hz, 1F, F^5), –137.8 (d, ^3J(F^3, F^2) = 20 Hz, 1F, F^3), –147.2 (ddd, ^3J(F^2, F^3) = 20 Hz, ^4J(F^2, H^6) = 6 Hz, ^5J(F^2, F^5) = 15 Hz, 1F, F^2).

GC-MS (M^+/retention time, min): 226/12.18 and 226/12.39 ($C_{12}H_6F_4$), 284/20.66, 284/20.93 ($C_{18}H_{11}F_3$).

HRMS (ESI) (mixture of $C_{12}H_6F_4$ and $C_{18}H_{11}F_3$), *m/z*: calcd. for $C_{12}H_6F_4$ 226.0405 and for $C_{18}H_{11}F_3$ 284.0813; found 226.0400 and 284.0809.

7. Attempted reaction of K[$C_6F_5BF_3$] with phenylethynyllithium

7.1. A three-necked flask equipped with a reflux condenser topped with bubbler and gas inlet/outlet tube, a Teflon-coated magnetic bar, septum inlet, and adapter for addition of PhC≡CLi was flushed with dry argon and charged with K[$C_6F_5BF_3$] (112 mg, 0.40 mmol) and DME (3 mL). Colorless solution was stirred for 10–15 min. PhC≡CLi in ether (0.40 M, 2 mL, 0.80 mmol) was siphoned into a measuring vessel and added in a one portion to above solution. The solution was stirred at 22 °C for 17 h and saturated aqueous solution of KF (6 mL) was added into flask. After 1 h an organic phase was decanted and dried with $MgSO_4$. The ^{19}F NMR spectrum showed signals of K[$C_6F_5BF_3$] (0.37 mmol) only.

7.2. Solution of K[$C_6F_5BF_3$] (121 mg, 0.44 mmol) in DME (3 mL) was reacted with PhC≡CLi in ether (0.40 M, 2 mL, 0.80 mmol) at 40 °C (bath) over a period of 2 h and treated as above. Starting K[$C_6F_5BF_3$] (0.36 mmol) was detected with ^{19}F NMR spectroscopy beside trace of unknown products.

7.3. Solution of K[$C_6F_5BF_3$] (123 mg, 0.44 mmol) in DME (4 mL) and PhC≡CLi in ether (0.45 M, 2 mL, 0.90 mmol) were combined, refluxed (58 °C, bath) for 5 h and cooled. A probe showed signals of **1-K** only (^{19}F NMR). The solution was treated with saturated aqueous solution of KF (10 mL) (see 7.1). The ^{19}F NMR spectrum showed signals of K[$C_6F_5BF_3$] (0.44 mmol).

8. Reaction of M[$C_6F_5BF_3$] with alkali halides

8.1. K[$C_6F_5BF_3$] (565 mg, 2.06 mmol) was added to solution of anhydrous LiBr (172 mg, 2.0 mmol) in MeCN (18 mL). Immediately white suspension formed. It was stirred for 1 h, centrifuged, and the mother liquor was evaporated to dryness on evaporator to give pale-yellow solid (668 mg; the expected yield for Li[$C_6F_5BF_3$]·2CH_3CN is 648 mg). Dissolution of a probe (0.12 mmol) in DME showed replacement of CH_3CN (0.22 mmol) with DME (see ^{11}B and ^{19}F NMR spectra, Table 4).

Li[$C_6F_5BF_3$]. ^{1}H NMR (DME): δ 3.42 and 3.26 (DME), 1.93 (non-coordinated CH_3CN) (0.22 mmol).

8.2. K[$C_6F_5BF_3$] (116 mg, 0.42 mmol) was added to stirred suspension of anhydrous LiI (173 mg, 1.29 mmol) in DME (1 mL) and ether (2 mL) and maintained over a period of 4 h. Li[$C_6F_5BF_3$] was obtained in quantitative yield (^{19}F NMR).

Li[$C_6F_5BF_3$]. ^{19}F NMR (DME–ether): δ –134.3 (m, 2F, $F^{2,6}$), –138.62 (q (1:1:1:1), ^{1}J(F, B) = 44 Hz, 3F, BF_3), –161.1 (t, $^{3}J(F^4, F^{3,5})$ = 20 Hz, 1F, F^4), –166.0 (m, 2F, $F^{3,5}$).

8.3. K[$C_6F_5BF_3$] (97 mg, 0.35 mmol) was added to suspension of anhydrous LiI (124 mg, 0.92 mmol) in DME (1 mL) and stirred at 70–73 °C (bath) for 5 h. Suspension was cooled and stirred with saturated aqueous KF for 2 h and organic phase was decanted. It contained K[$C_6F_5BF_3$] (0.06 mmol) and C_6F_5H (0.25 mmol).

8.4. $K[C_6F_5BF_3]$ (155 mg, 0.56 mmol) was added to suspension of anhydrous LiBr (148 mg, 1.7 mmol) in DME (2 mL) and stirred at 55–60 °C (bath) for 4 h. After cooling, the supernate contained $Li[C_6F_5BF_3]$ (0.44 mmol), $C_6F_5B(OH)_2$ (0.06 mmol) and C_6F_5H (0.03 mmol).

8.5. $K[C_6F_5BF_3]$ (156 mg, 0.56 mmol) was added to suspension of anhydrous LiCl (90 mg, 2.0 mmol) in DME (2 mL) and stirred at 55–60 °C (bath) for 4 h. After cooling, the supernate contained $Li[C_6F_5BF_3]$ (0.39 mmol), $C_6F_5B(OH)_2$ (0.07 mmol) and C_6F_5H (0.03 mmol).

8.6. Suspension of $Li[C_6F_5BF_3]$ (0.25 mmol) and anhydrous LiI (53 mg, 0.40 mmol) in DME (0.5 mL) was kept at 65–70 °C (bath) for 4 h to form a brownish solution. It was washed with saturated aqueous KF and organic phase was decanted. It contained $K[C_6F_5BF_3]$ (0.02 mmol, 91% conversion) and C_6F_5H (0.22 mmol).

8.7. $[Bu_4N][C_6F_5BF_3]$ (0.4 mmol) and anhydrous LiI (94 mg, 0.70 mmol) in diglyme (0.5 mL) was stirred at 65–70 °C (bath) for 4 h. After centrifugation the organic phase was decanted, precipitate was washed with diglyme. The combined solution was washed with saturated aqueous KF and analyzed by the ^{19}F NMR spectroscopy to show presence of $[C_6F_5BF_3]^-$ (0.17 mmol, 57% conversion) and C_6F_5H (0.23 mmol).

9. Stability of $M[C_6F_5BF_3]$ in solution

9.1. Solution of $Li[C_6F_5BF_3]$ in DME was kept at 22 °C for 1 week. No reaction occurred (^{19}F NMR). Heating in a sealed tube at 60–68 °C for 5 h gave the same result.

9.2. Solution of $[Bu_4N][C_6F_5BF_3]$ in diglyme was kept in a sealed tube at 60–68 °C for 4 h. No changes occurred.

10. Reaction of $K[C_6F_5BF_3]$ with MeONa (excess)

A 20 mL flask equipped with a magnetic stir bar was charged with DMF (5 mL) and MeOH (111 mg, 3.4 mmol). NaH (119 mg, 2.8 mmol) was added in one portion to cause gas evolution and foaming. Suspension was stirred at 25 °C for 1 h under an atmosphere of argon and $K[C_6F_5BF_3]$ (259 mg, 0.94 mmol) was added in one portion.

The flask was immersed into a pre-heated oil bath (130 °C), and the reaction mixture was stirred for 5 h. The ^{19}F NMR spectrum showed the formation of M[4-$MeOC_6F_4BF_3$], M[3,4-$(MeO)_2C_6F_3BF_3$], M[2,4-$(MeO)_2C_6F_3BF_3$] and 2,3,5,6-C_6F_4HOM (M = K, Na) in ratio 100 : 15 : 19 : 24. Water (0.5 mL) and K[HF_2] (0.9 g) were added, and mixture was stirred at 25 °C for 1 h. Organic phase was decanted and evaporated under reduced pressure. Residue was washed with CH_2Cl_2 (2 × 8 mL), dried on evaporator and extracted with DME (2 mL). Solution contained K[4-$MeOC_6F_4BF_3$] [10] (0.60 mmol), K[3,4-$(MeO)_2C_6F_3BF_3$] (**22**) (0.10 mmol), K[2,4-$(MeO)_2C_6F_3BF_3$] (**23**) (0.13 mmol) and 2,3,5,6-C_6F_4HOH [10] (0.03 mmol) (^{19}F NMR, with CF_3COOH quantitative internal reference).

K[3,4-$(MeO)_2C_6F_3BF_3$] (22). ^{19}F NMR (DME): δ –130.4 (dq (1:1:1:1), ^5J(F^2, F^5) = 12 Hz, ^4J(F^2, B*F*) = 12 Hz, 1F, F^2), –137.8 (m, 3F, BF_3), –138.6 (dq (1:1:1:1), ^3J(F^6, F^5) = 24 Hz, ^4J(F^6, B*F*) = 12 Hz, 1F, F^6), –163.2 (dd, ^3J(F^5, F^6) = 24 Hz, ^5J(F^5, F^2) = 12 Hz, 1F, F^5). ^{19}F NMR (DMF): δ –127.2 (dq (1:1:1:1), ^5J(F^2, F^5) = 12 Hz, ^4J(F^2, B*F*) = 12 Hz, 1F, F^2), –132.8 (m, 3F, BF_3), –135.8 (dq (1:1:1:1), ^3J(F^6, F^5) = 24 Hz, ^4J(F^6, B*F*) = 12 Hz, 1F, F^6), –161.5 (dd, ^3J(F^5, F^6) = 24 Hz, ^5J(F^5, F^2) = 12 Hz, 1F, F^5).

K[2,4-$(MeO)_2C_6F_3BF_3$] (23). ^{19}F NMR (DME): δ –137.8 (m, 3F, BF_3), –138.4 (ddq (1:1:1:1), ^3J(F^6, F^5) = 24 Hz, ^5J(F^6, F^3) = 12 Hz, ^4J(F^6, B*F*) = 12 Hz, 1F, F^6), –154.7 (d, ^5J(F^3, F^6) = 12 Hz, 1F, F^3), –161.3 (d, ^3J(F^5, F^6) = 24 Hz, 1F, F^5). ^{19}F NMR (DMF): δ –132.8 (m, 3F, BF_3), –135.5 (ddq (1:1:1:1), ^3J(F^6, F^5) = 24 Hz, ^5J(F^6, F^3) = 12 Hz, ^4J(F^6, B*F*) = 12 Hz, 1F, F^6), –153.3 (d, ^5J(F^3, F^6) = 12 Hz, 1F, F^3), –160.4 (m, 1F, F^5).

References

1. Adonin, N. Y.; Babushkin, D. E.; Parmon, V. N.; Bardin, V. V.; Kostin, G. A.; Mashukov, V. I.; Frohn, H.-J. *Tetrahedron* **2008,** *64*, 5920-5924.
2. Tegnér, C. *Acta Chem. Scand.* **1952,** *6*, 782-790.
3. Jones, R. G.; Gilman, H. The Halogen-Metal Interconversion Reaction with Organolithium Compounds. In *Organic Reactions;* Adams, R., Ed.; Vol. 6; John Wiley & Sons, Inc.: New York, 1951; pp 339-356.
4. Eaborn, C.; Walton, D. R. M. *J. Organomet. Chem.* **1964,** *2*, 95-97.
5. Watson, S. C.; Eastham, J. F. *J. Organomet. Chem.* **1967,** *9*, 165-168.
6. Adonin, N. Y.; Shabalin, A. Y.; Bardin, V. V. *J. Fluorine Chem.* **2014,** *168*, 111-120.

7. Bogachev, A. A.; Kobrina, L. S. *Zhurnal Org. Khimii* **1997,** *33*, 742-744. ; *Russ. J. Org. Chem.* **1997,** *33*, 681-683.
8. Bolton, R.; Sandall, J. P. B.; Williams, G. H. *J. Fluorine Chem.* **1974,** *4*, 355-361.
9. Wei, Y.; Kan, J.; Wang, M.; Su, W. P.; Hong, M. C. *Org. Lett.* **2009,** *11*, 3346-3349.
10. Shabalin, A. Y.; Adonin, N. Y.; Bardin, V. V.; Prikhod'ko, S. A.; Timofeeva, M. N.; Bykova, M. V.; Parmon, V. N. *J. Fluorine Chem.* **2013,** *149*, 82-87.
